# Case report: Total percutaneous post-closure of femoral arterial access sites after veno-arterial extracorporeal membrane oxygenation

**DOI:** 10.3389/fmed.2022.980122

**Published:** 2022-09-15

**Authors:** Lun Tian, Libin Zhang, Naiding Zhang, Xin Xu, Yongshan Xu, Zhenjie Liu, Man Huang

**Affiliations:** ^1^Department of Vascular Surgery, The Second Affiliated Hospital of Zhejiang University School of Medicine, Hangzhou, China; ^2^Department of Intensive Care Unit, The Second Affiliated Hospital of Zhejiang University School of Medicine, Hangzhou, China

**Keywords:** Perclose Proglide, arteriotomies, VA-ECMO, suture-mediated closure device, integrated algorithm

## Abstract

Veno-arterial extracorporeal membrane oxygenation (VA-ECMO) which is a form of circulatory and gas exchange support. Following VA-ECMO, total percutaneous closure of the site of femoral arterial puncture with perclose Proglide (PP) has become widespread, enhancing patient comfort and lessening the incidence of wound infections and lymphatic fistulas in a short closure time. The preclose technique with perclose Proglide provides numerous benefits, however, it prolongs extra time during the VA-ECMO procedure, adds additional post-operative care to workloads, and increases the potential for Proglide stitch infection. The modified technique-percutaneous post-closure, described here by a case of a 65-year-old man with heart attack who underwent VA-ECMO, is a simple, rapidly applied technique to wean VA-ECMO also suitable for emergency cannulation. The patient was administered mechanically ventilated and sedated and the femoral artery access site and evaluated by ultrasound for precise positioning, then the VA-ECMO arterial cannula was withdrawn, and a 0.035-in guidewire was left in the artery. The first set of sutures was deployed after the Proglide device was inserted over the guidewire. The second sutures were then replaced in the same way but at a different angle. After hemostasis was achieved, the guidewire was removed, and additional manual compression was used to control any residual blood seeping. No hematoma, pseudoaneurysm, major bleeding, minor bleeding, acute arterial thrombosis, arteriovenous fistula, groin infection, lymphocele, or arterial dissection and stenosis occurred during the periprocedural period or during the 30-day post-procedural follow-up. In conclusion, the standardized algorithm we established, total percutaneous post-closure of femoral arteriotomies utilizing Perclose ProGlide device is feasible and safe with a low incidence of access site complications.

## Introduction

Veno-arterial extracorporeal membrane oxygenation (VA-ECMO) may be of benefit as salvage therapy in select patients who continue to have unstable circulatory and profound gas-exchange abnormalities, which provided encouraging outcomes regarding the efficacy and economic assessment of this strategy (1–3). In general, the common size sheath of 14–20 F is used for ECMO insertion, hemostatic stitches or prolonged manual compression can be used to achieve arteriotomy hemostasis, while large arteriotomy wounds require surgical vascular repair as a standard weaning strategy. As vascular complications following decannulation, to improve patient outcomes after percutaneous decannulation, cannula removal techniques, particularly a standardized procedural technique with a suitable closure device are essential. The ProGlide (4) (Abbott Vascular Inc., Redwood, California) is a suture-based closure device that deploys a suture on either side of the arterial wall at the arteriotomy site, mimicking open surgical closure, designed to access created by large-bore catheters, stopping the blood flow. Since it is a mechanical closure, there are theoretically no limitations to re-access or the administration of antiplatelet and anticoagulant. The ProGlide can track over a standard 0.035-inch (or smaller) wire and accommodate 5- to 21-Fr. arteriotomies, but with a larger than 8-Fr. sheath, at least two devices are required to close the arteriotomy. Therefore, the device facilitates various vascular interventions and the Proglide deployed prior to the insertion of the large bore devices is often referred as “Perclose technique.” The preclose technique with perclose Proglide (PP) allows for rapid arteriotomy closure after percutaneous interventions as it is used immediately once the procedure has concluded. They allow early ambulation and discharge after groin puncture and mitigate patient discomfort from extended manual compression (5, 6). Proglide-assisted closure devices in preclosure have been popular proposed and applied to VA-ECMO decannulation (6–9), which minimize invasiveness, shorten procedure time, release post-operative pain, and decrease the risk of wound complications. However, despite the obvious benefits, they have not gained universal adoption among interventionalists, as it prolongs extra time during the VA-ECMO procedure, adds additional post-operative care to workloads, and increases the potential for ProGlide stitch infection. Primarily based on shortcomings or defects, the modified technique has been proposed—percutaneous post-closure. We described a case of percutaneous post-closure for VA-ECMO decannulation, which revealed this technique under the evaluation of ultrasound safely and successfully closed arterial ECMO decannulation sites after arteriotomy wound closure with improved accuracy.

## Case description

A 65-year-old man with a myocardial infarction leading to cardiac arrest who underwent VA-ECMO and intra-aortic balloon pumping was deployed bedside percutaneous decannulation by Perclose ProGlide (Abbott Vascular Inc., USA) at the arteriotomy site. The patient was sedated and mechanically ventilated, and local anesthesia was administered at the femoral artery access site. The following steps detail the implementation sequence to close the arteriotomy of VA-ECMO decannulation using two Perclose ProGlide devices.

### Preparation phase

**Figure 1:**
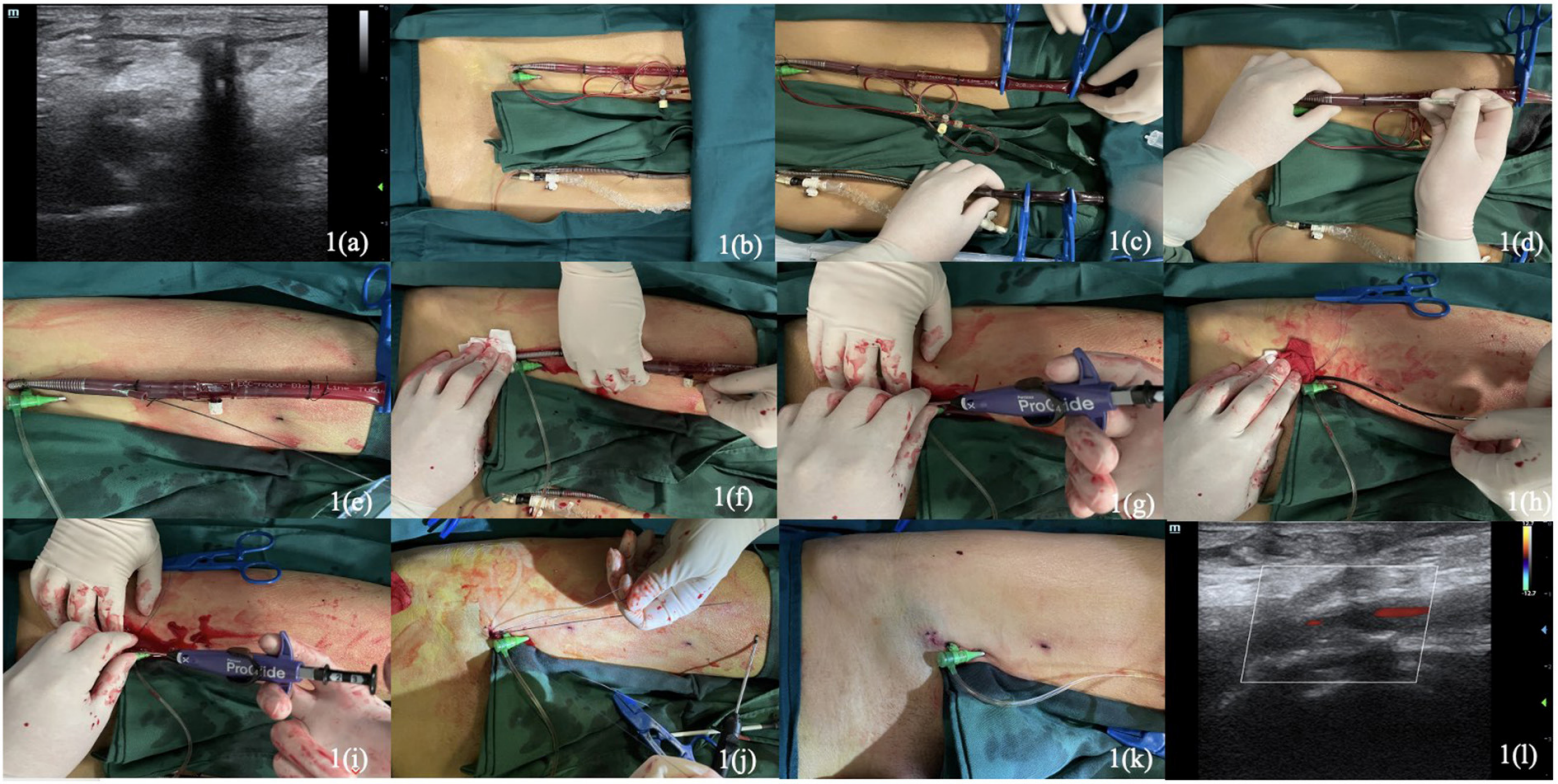
Pre-operative ultrasound images **(a)**. Sterile preparation of the operative area **(b)**. The clamping of arterial and venous cannulas at the distal part by four tubing clamp forceps **(c)**. Direct puncture with seldinger technique in the proximal portion of the arterial cannula **(d)**. The insertion of Terumo Stiff guidewire through the cannula **(e)**. Removal of the arterial cannula under manual compression at the access by an assistant **(f)**. Insertion of the first Perclose Proglide and deploying at the direction about 10–11 o' clock **(g)**. Removal of the first Proglide and placement a clamp forcep to hold the two suture limbs together on the right side of the patient **(h)**. Reinsertion of the guidewire to place the second Proglide and deploy at about 1–2 o' clock **(i)**. Wrapping the railed suture limbs tightly around the left index finger to pull it **(j)**. The hemostasis was adequate and cut the suture tails **(k)**. Post-operative ultrasound images with normal blood flow of femoral artery **(l)**.

Pre-operative imaging should be closely scrutinized for anatomic information regarding the arterial sheath catheter, common and superficial femoral artery, including its diameter, associated occlusive disease, particularly anterior calcification, and the location of the femoral bifurcation relative to the femoral head and inguinal ligament, record Doppler ultrasonic blood flow signal simultaneously. Therefore, femoral artery ultrasound was estimated (Figure 1a) before catheter decannulation and marked when necessary. Then thoroughly sterile preparation for the cannulation area was in place (Figure 1b).

### Procedural description

Above all, the arterial and venous cannulas were instantly clamped at the distal part with four tubing clamp forceps (Maquet, Germany, 20 cm) (Figure 1c) before being cut with a scissor. After direct puncture with seldinger technique (Figure 1d) in the proximal portion of the arterial cannula (Maquet, Germany, 23 cm, 19 F), a 0.035-inch guidewire (Terumo Stiff Wire, Terumo, Japan, 150 cm) was advanced cautiously into to ascending aorta (Figure 1e), the VA-ECMO arterial cannula was withdrawn and removed under manual compression at the access by an assistant (Figure 1f). Subsequently, the first Perclose Proglide was inserted through the guidewire and continued to advance the device until a vigorous pulsating flow of blood was plainly obvious from the marker lumen, placed at about 10–11 o' clock (Figure 1g), then deployed the foot by lifting the lever (marker #1), gently pulled the device back, using the other hand to push on the plunger assembly (as shown in marker #2), disengaged the needles by pulling the plunger assembly back (as shown in marker #3) and dislodged the plunger and needles utterly. Pulled back the plunger until the suture was totally taut, then used the QuickCuts to cut the suture. Pushing the lever (marker #4) down to the main body to return the foot to its premier closed position, releasing the suture knot, and continuing to remove the device until the guide wire was visible while holding the two suture limbs pull through the distal end of the proximal guide. Placed a clamp forcep instantly to hold the two suture limbs together on the patient's right side. Reinserted the guidewire (Check-Flo Performer, Cook Medical, USA) maintaining an adequate length inside the artery and removed the first ProGlide (Figure 1h). Repeat aforementioned steps with the second ProGlide, which is placed at about 1–2 o' clock (Figure 1i) toward the patient's left side, and the other clamped suture were placed on the left side. Advanced the guidewire into the artery and irrigated the sutures with heparinized saline. Removed the clamp from the first suture, applied manual pressure proximal to the puncture site and wrapped the railed suture limb tightly around the left index finger to pull it (Figure 1j). Removed the clamp from the second suture and advance the knot using the same technique while ensuring guidewire access. The bleeding was controlled, and the hemostasis was adequate, ultimately cutting the suture tails below the surface of the skin (Figure 1k).

After completing the procedure, we assessed the incision by ultrasound, the blood flow of femoral arterial signals was well-filled with no abnormal signals outside the blood vessels (Figure 1l). Manual compression was continued for at least 5 min, and the puncture site was covered with a pressure bandage for 12 h. Another Doppler ultrasound control was performed 3 days later to exclude hematoma, arteriovenous fistula, or pseudoaneurysm after release of the pressure bandage and estimated again 1 month later. The double Proglide-assisted post-closure technique was successful with adequate hemostasis without extra endovascular or surgical procedures to prevent vessel blood leaking. The hemostatic good control with no sign of access-related complications and other adverse events, such as massive bleeding, acute limb ischemia, lymphocele, groin infection, vessel dissection, occlusion, any stenosis or arterial thrombosis during the periprocedural period, defined as arterial closure within 48 h and 30-day follow-up. More detailed clinical relative information, data, and parameters can be achieved from medical record review during ECMO-inserted.

## Discussion

Veno-arterial extracorporeal membrane oxygenation has emerged as the preferred treatment option for patients suffering from cardiogenic shock or cardiac arrest. Regarding ECMO weaning, diverse strategies have been described for achieving hemostasis from cannula removal. Simple manual compression is effective in small puncture sites (4–8 F) in the majority of cases. The advent of percutaneous technique, especially the Perclose Proglide, the most common vascular closure device, decreased the morbidity of the procedure, minimized the need for compression, address patient complaints, and significantly shortened the bedrest period even further. A prospective randomized study looking at the Perclose Proglide device compared with manual compression showed that there was shorter time to hemostasis, ambulation, and discharge with suture mediated closure, with no difference in overall complication rate between the two treatment groups (10). Practitioners have used the ProGlide device to close much larger puncture sites by placing two Proglide devices through a single puncture site, thus allowing a totally percutaneous method to wean VA-ECMO, which is referred as standard “preclose technique.” This technique has been verified to be a convenient, safe, and effective procedure for repairing arteriotomy sites in quite a bit of procedures (11), including endovascular aortic repair (EVAR) and other percutaneous treatments. These devices may increase the risk of groin infection and leg ischemia but decrease the time needed to achieve hemostasis (4).

**Figure 2:**
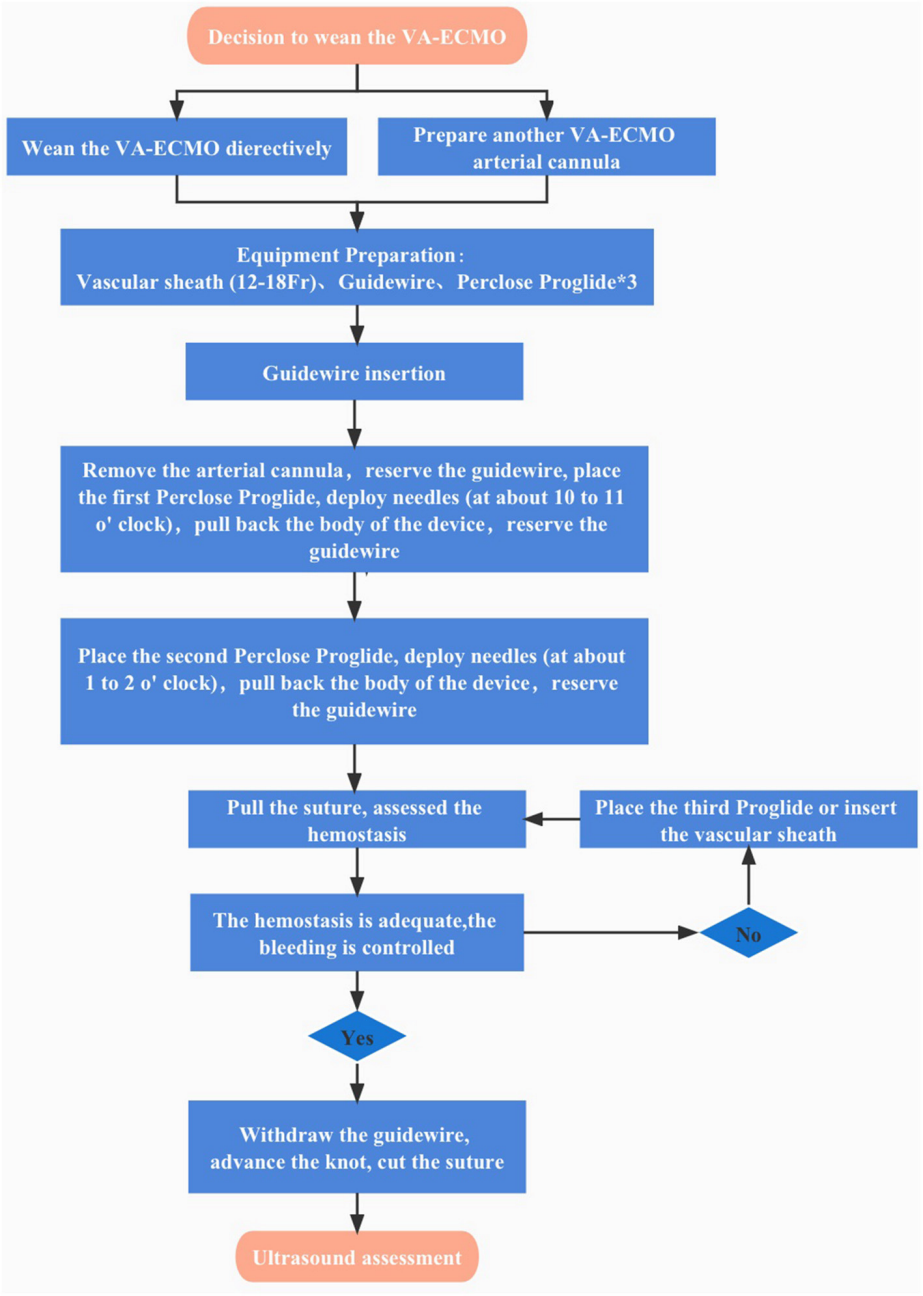
Algorithm of total percutaneous post-closure of femoral arterial access sites after Veno-arterial Extracorporeal Membrane Oxygenation.

Revolutionary precepts using Proglide-assisted closure devices in preclosure have been popular proposed and applied to VA-ECMO decannulation (6, 7). Total percutaneous suture arteriotomy wounds minimize invasiveness compared with femoral cutdown access repair. A most recent single-center study using Perclose ProGlide suture-mediated system with preclose technique shows satisfactory successful closure rates with fewer limb complications and bleeding events and a reduction in total operative time without increasing hospital stay length (5). While we propose that potential defects are the following: (1) Preclose technique increases operational complexity and likelihood of error during VA-ECMO implantation of cannulation and prolongs the whole operating time, which means operators should be adequately strictly trained. There is a learning curve with this device, despite the high technical success rates observed in recent series; (2) Pre-operative imaging for anatomic information must be scrutinized, and small access vessel diameter and anterior wall calcification will become factors of failure, which undoubtedly infeasible in emergency rescue; (3) the Proglide system stitches remain in the large arteriotomy access which may be more vulnerable to infection in the prolonged time, considering the uncertain duration of extracorporeal therapy; (4) the Proglide suture adds extra post-operative care burden of medical staff, tearing, injury, dissection of vessels may occur during repeated care operations. Given several reasons mentioned above, we put forward the innovative techniques and protocols in post-closure technique applying to VA-ECMO decannulation (Figure 2). Some experts are hesitant to the effectiveness and safety reliability of post-closure of large-bore arterial access, it has been suggested that post-closure may increase technical difficulties during VA-ECMO decannulation procedure and be prone to excessive blood loss (6). We further modified this protocol inserting the guidewire by a direct puncture technique without cutting down the arterial catheter. In this case, we successfully wean the VA-ECMO and decannulate the arterial catheter *via* post-closure technique within 10 min coordinated by two experienced vascular surgeons, under routine use of ultrasound guidance to increase the accuracy of arterial access which achieved a shorter procedure time, less post-operative pain, and a lowered risk of wound complications.

The decision to use a closure device on a given access site should be individualized to the patient's anatomy, clinical circumstances, and likelihood of benefit. For a safe and successful decannulation of ECMO catheter, it is critical that high-risk vessels be identified on either pre-operative imaging or initial angiography. Therefore, this may prompt earlier anticoagulation, use of protection devices, or use of smaller catheters and sheaths to prevent adverse outcomes. Moreover, manual compression is commonly held between 5 and 20 min after percutaneous closure device depending on the extent of anticoagulation, the puncture size, and the patient's blood pressure to make sure adequate hemostasis. Overall, this complete percutaneous post-closure perspective of arteriotomies provides an effective, safe, and simple practice for the management of patients facilitating VA-ECMO decannulation even in patients with poor vascular conditions.

## Data availability statement

The original contributions presented in the study are included in the article/supplementary material, further inquiries can be directed to the corresponding author/s.

## Ethics statement

The studies involving human participants were reviewed and approved by the Ethics Committee of The Second Affiliated Hospital of Zhejiang University, School of College. The patients/participants provided their written informed consent to participate in this study.

## Author contributions

LT gleaned the patient's detailed information, took the relevant pictures, and compoesd the manuscript. ZJL performed the surgical procedures. NDZ and XX underwent data curation and validation. ZJL reviewed and edited the original draft. All authors contributed to the article and the final manuscript was approved by all of them.

## Funding

This work was provided funding with the National Natural Science Foundation of China (81670433 and 81970398), the Project of Zhejiang Medical Young Talents (2017), Zhejiang Medical and Health Science and Technology Project (2020RC014), and the Natural Science Foundation of Zhejiang Province (Q20H020059). Science Fund for Distinguished Young Scholars of Zhejiang Province (LR22H020002). Natural Science Foundation of Zhejiang Province (LQ20H020008).

## Conflict of interest

The authors declare that the research was conducted in the absence of any commercial or financial relationships that could be construed as a potential conflict of interest.

## Publisher's note

All claims expressed in this article are solely those of the authors and do not necessarily represent those of their affiliated organizations, or those of the publisher, the editors and the reviewers. Any product that may be evaluated in this article, or claim that may be made by its manufacturer, is not guaranteed or endorsed by the publisher.
